# Prevalence of vitamin B12 deficiency, associated factors, and correlation with CD4 count among HIV-positive adults at Kayunga Regional Referral Hospital, Central Uganda

**DOI:** 10.1186/s12981-025-00791-z

**Published:** 2025-09-29

**Authors:** Mohamed Jayte, Mai Abdalla Ali, Abdifatah Hersi Karshe, Abdifitah Abdullahi Mohamed, Farah Dubad Abdi, Yahye Mohamed Jama, Theoneste Hakizimana, Awil Abdulkadir Abdi, Abukar Ali Ahmed, Abishir Mohamud Hirsi

**Affiliations:** 1 Department of Internal Medicine, Faculty of Clinical Medicine and Dentistry, Kampala International University, Ishaka, Bushenyi, Uganda; 2 Department of Microbiology and Immunology, Faculty of Biomedical Sciences, Kampala International University, Ishaka, Bushenyi, Uganda; 3 Department of Obstetrics and Gynecology, Faculty of Clinical Medicine and Dentistry, Kampala International University, Ishaka, Bushenyi, Uganda

**Keywords:** Vitamin B12, HIV, Deficiency, Uganda

## Abstract

**Background:**

Vitamin B12 deficiency is a common but under-recognized comorbidity among HIV-infected individuals, contributing to anemia, neurological impairment, and poor immune recovery. In sub-Saharan Africa, where HIV burden is high, routine screening for B12 deficiency is rarely performed, and data in Uganda are scarce. This study aimed to determine the prevalence of vitamin B12 deficiency, identify associated factors, and examine its correlation with CD4 count among HIV-positive adults.

**Methods:**

We conducted a cross-sectional study among 156 HIV-positive adults at Kayunga Regional Referral Hospital, Uganda. Serum vitamin B12 was measured using the ARCHITECT B12 assay. Deficiency was defined as < 200 pg/mL. Logistic regression and Spearman correlation were used to identify predictors and assess relationships with CD4 counts.

**Results:**

Vitamin B12 deficiency was present in 25% of participants. Significant independent predictors included: low income (aOR 2.5, 95% CI 1.07–5.75), ART-naïve status (aOR 2.9, 95% CI 1.03–8.73), underweight BMI (aOR 4.2, 95% CI 1.89–9.60), and HIV duration > 10 years (aOR 4.0, 95% CI 1.32–12.1). CD4 count showed a modest inverse correlation (ρ = − 0.24, *p* < 0.001).

**Conclusion:**

Vitamin B12 deficiency is prevalent among HIV-positive adults in Uganda. Routine screening and nutritional interventions are recommended, especially for high-risk groups.

## Background

Vitamin B12 deficiency significantly affects HIV-infected individuals, exacerbating immunosuppression and negatively influencing treatment outcomes [1, 2]. Despite documented risks, routine screening remains uncommon in Uganda [2]. Vitamin B12 deficiency has significant health implications including anemia, neurological dysfunction, and compromised immune response [3, 4]. Prevalence globally varies from 5.4% in Italy to as high as 47.05% in India among HIV-infected populations, reflecting demographic and nutritional disparities [5–7]. Sub-Saharan Africa, home to over 25 million HIV-infected individuals, has limited data available on the prevalence, determinants, and clinical impact of vitamin B12 deficiency in HIV-positive populations. Nigerian studies reported a 29.3% deficiency among ART-naïve patients, highlighting regional nutritional challenges [8]. Ugandan studies previously reported lower deficiency prevalence rates of approximately 10.3% among ART-naïve HIV-positive adults, but data for individuals actively receiving ART remains sparse [2]. This study aimed to determine vitamin B12 deficiency prevalence, identify associated factors, and assess correlation with CD4 counts among HIV-positive adults attending Kayunga Regional Referral Hospital (KRRH), Central Uganda.

## Methods

A cross-sectional study was conducted among 156 HIV-positive adults at the KRRH HIV clinic between January and June 2024. Participants were recruited using systematic random sampling from the HIV clinic appointment register until the target sample size was achieved. The sample size was calculated using the Kish–Leslie formula, based on a previous prevalence of 10.3% from Semeere et al. [9], with adjustments for non-response. Eligible participants were adults aged ≥ 18 years who provided informed consent. Exclusion criteria included vitamin B12 or multivitamin supplementation within the past 3 months, pregnancy, liver cirrhosis, or hematologic malignancy. Pregnancy status, liver cirrhosis, and hematologic malignancy were confirmed through review of medical records and/or clinician diagnosis documented in the patient file. Vitamin B12 deficiency was defined as serum levels < 200 pg/mL using the ARCHITECT B12 assay. Structured questionnaires captured sociodemographic, clinical, and nutritional data, and the most recent CD4 count within the preceding 6 months was retrieved from the patient’s medical records. Logistic regression was used to identify predictors of vitamin B12 deficiency, and Spearman correlation explored the relationship between CD4 counts and vitamin B12 levels. Ethical approval was granted by Kampala International University Ethics Committee (Ref: KIU-2024-540).

## Results

### Participant characteristics

Of the 156 HIV-positive adults enrolled, the mean age was 39.6 ± 9.4 years, and 53.8% were female. Most participants were under 29 years old (73.1%), unemployed (54.5%), and residents of Kayunga district (61.5%). Educational attainment was generally low, with 48.1% completing primary education and 32.1% secondary. Nearly half (44.9%) reported low monthly income. Alcohol consumption and smoking were reported by 28.2% and 20.5% of participants, respectively.

From a clinical perspective, the majority had normal BMI (62.8%), though 10.9% were underweight and 9.6% obese. Most were receiving antiretroviral therapy (84.6%), primarily the TDF/3TC/DTG regimen (79.5%). CD4 counts ranged between 200 and 499 cells/mm³ in 46.8% of participants, while 12.2% had CD4 < 200. Over half (51.9%) were in WHO HIV Stage 1, and 22% had lived with HIV for more than 10 years.

### Prevalence of vitamin B12 deficiency among HIV attending at KRRRH

**Fig. 1:**
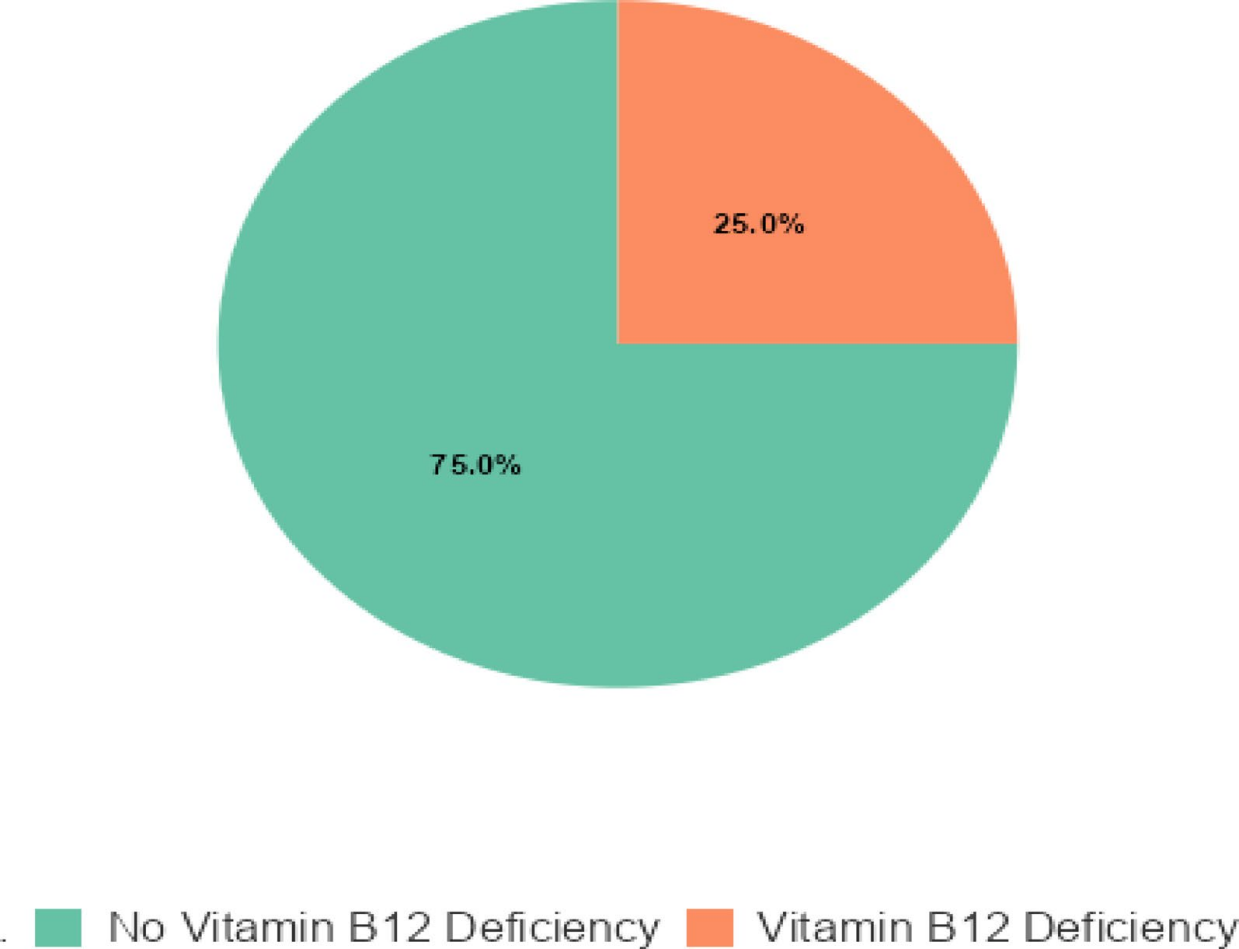
Showinf Prevalance of Vitamin B12 deficiency among Hiv

Among the 156 participants, Vitamin B12 deficiency was identified in 39 participants, indicating a prevalence of 25% as showing Fig. 1 below.

## Factors associated with vitamin B12 deficiency

The bivariable analysis identified several factors associated with vitamin B12 deficiency at *p* < 0.05, including male gender (*p* = 0.026), low monthly income (*p* = 0.004), ART-naïve status (*p* < 0.001), and HIV duration > 10 years (*p* < 0.001). Among these, variables with *p* < 0.02 (low monthly income, ART-naïve status, and HIV duration > 10 years) were selected for multivariable analysis. BMI (< 18.5) (*p* = 0.108) was also included in the multivariable model despite not reaching statistical significance. In the adjusted analysis, low monthly income showed 2.5 times higher odds of deficiency compared to high income, ART-naïve status demonstrated 2.9 times greater odds than those on ART, underweight BMI had 4.2 times increased odds versus normal BMI, and HIV duration > 10 years showed 4 times higher odds relative to duration ≤ 10 years. The association with male gender observed in bivariable analysis was no longer significant after adjustment (*p* = 0.098). These results identify key independent predictors of vitamin B12 deficiency in this population as showing in Table 1.

**Table 1:** Bivariable and multivariable logistic regression of factors associated with vitamin B12 deficiency among HIV-positive adults (*N* = 156)

Variable	Bivariable analysis crude OR (95% CI)	*p*-value	Multivariable analysis adjusted OR (95% CI)	*p*-value	Reference category
*Gender*					Female
Male	2.3 (1.10–4.80)	0.026	1.9 (0.88–4.10)	0.098	
*Monthly income*					High income
Low income	4.5 (1.61–13.07)	0.004	2.5 (1.07–5.75)	0.032	
*ART status*					On ART
ART-naïve	6.4 (2.86–14.35)	< 0.001	2.9 (1.03–8.73)	0.044	
*BMI (kg/m²)*					≥ 18.5
< 18.5	2.4 (0.82–7.09)	0.108	4.2 (1.89–9.60)	< 0.001	
*Duration of HIV infection*					≤ 10 years
> 10 years	7.0 (2.81–17.60)	< 0.001	3.99 (1.32–12.06)	0.014	

Reference categories—Gender: Female; BMI: ≥18.5 kg/m²; Monthly income: High income; ART status: On ART; Duration of HIV infection: ≤10 years. Variables with *p* < 0.2 in bivariable analysis were entered into the multivariable model

### Correlation between CD4 cell count and Vitamin B12 deficiency among HIV patients attending KRRH (N = 156)

An inverse correlation between CD4 counts and vitamin B12 deficiency was also observed (Spearman’s rho = − 0.24; *p* < 0.001).

## Discussion

The observed prevalence (25%) closely mirrors findings from Nigeria (29.3%), indicating shared regional nutritional and socioeconomic challenges [8, 10, 18, 19]. ART-naïve status was significantly associated with deficiency, likely due to untreated HIV-related inflammation and mucosal damage impairing absorption, consistent with prior literature [2, 10]. The strong association with low BMI underscores underlying nutritional deficiencies common among HIV patients with poor intake or gastrointestinal malabsorption [11, 15–17]. Longer HIV infection duration correlated with increased vitamin B12 deficiency, reflecting cumulative gastrointestinal impairment and heightened metabolic demands in advanced disease stages [2, 12–14]. The inverse correlation between CD4 counts and vitamin B12 levels aligns with existing evidence linking advanced immunosuppression and micronutrient deficiency [7, 9, 20, 21].

Cross-sectional and single-center design limit causality inference and generalizability; however, findings support targeted interventions for ART-naïve and long-duration HIV-infected individuals. Routine screening and nutritional management could significantly enhance outcomes.

## Conclusion

Vitamin B12 deficiency remains a common comorbidity among HIV-positive adults in Uganda, particularly among socioeconomically disadvantaged, ART-naïve, undernourished individuals, and those with long-standing HIV infection. These findings underscore the importance of incorporating routine vitamin B12 screening and nutritional interventions into HIV care protocols, especially in resource-limited settings. Integrating micronutrient assessment into clinical guidelines may enhance immune recovery and overall patient outcomes.

## Data Availability

The datasets used and/or analyzed during the current study are available from the corresponding author upon reasonable request.
